# Slipping of the Intraperitoneal Ligature

**Published:** 1899-08

**Authors:** R. Stansbury Sutton

**Affiliations:** Pittsburg, Pa.


### SLIPPING OF THE INTRAPERITONEAL LIGATURE.

By R. STANSBURY SUTTON, M. D., Pittsburg, Pa.

THE cause of the slipping of a ligature placed upon an exposed vessel is always due either to a careless application of the ligature, or to a giving way of the knot or both. The careless application of the ligature is not pardonable. The giving way of the knot, on a silk ligature, properly applied, I have never seen. When the ligature is one of catgut, however carefully it is applied to a vessel within the peritoneal cavity, the knot may slip. For this reason, for intraperitoneal ligation of several bloodvessels I prefer and use silk. But notwithstanding the reliability of the knot, on a properly tied silk ligature, when the ligation of tissue is effected en masse, the ligature will occasionally slip off the mass; or more properly expressed, a portion of the constricted mass, and notably that portion containing the artery, will retract from the embrace of the ligature and hemorrhage into the peritoneal cavity will ensue. In order to avoid this accident after abdominal section Professor Billroth, before applying a ligature en masse, applied with full force a pair of heavy clamp forceps and in the groove of crushed tissue tied the ligature. Where I have followed this example the ligature has never slipped. The treatment of hemorrhoids by crushing was established twenty years ago by Allingham and has stood the test of time. The fact that a crushed bloodvessel does not bleed was established for all, by such accidents as occur daily in railroad wrecks and rolling mills. The surgeon observed the fact and applied the deduced principle of crushing tissue to prevent bleeding to his art. The hemostatic forcep of Peán, the heavy clamp forceps of Billroth, the hemorrhoid crusher of Allingham. and the recently devised crusher of Doyen, are all efforts to apply systematically to surgery, the fact taught us by crushing accidents.

In my first 492 intraperitoneal operations, I encountered two cases of slipped ligature. The pedicles in both were short and thick and I neglected to crush a groove for the ligature. In both cases the collapse was profound and my own action was immediate in both cases. While ether was being given I reopened the abdominal wound, secured the bleeding vessel, and washed the lost blood and clots out of the cavity and reclosed the wound. I did one case by candle light, and immediately afterward transfused the patient. Both cases recovered, one occurring in January, 1895, and one a few months ago. In addition to these two cases of my own, I

know of six other cases, which have occurred in the hands of four other operators in this city, and not one of the six cases was saved. All died. Tying tissue en masse is not surgical, and as time goes on I endeavor more and more to avoid it. Short pedicles in instances of salpingo-oöphorectomy are not infrequent, and it is better surgery to tie the ovarian artery at the big infundibulum pelvicum, and the uterine below the cornual extremity of the Fallopian tube and to strip out the tube and ovary entirely from the ligamentum latum, closing the open edge of the latter with a catgut suture.

The conical, retracting stump is avoided; all tension is obviated, and the removal of the ovary and tubes more complete. Tying en masse is avoided, and secondary hemorrhage will not occur. Doyen's instrument will serve a useful purpose in all cases where it is not possible to safely secure vessels with the ligature. It is a very formidable looking affair, but well constructed and practical. But the convenience of an instrument so useful and convenient should not tempt operators to use it to the exclusion of sound surgical methods found to be reliable in their experience. Discarding the temptation to ligate en masse, and keeping before us the facts relative to the retraction of constricted tissues, the constantly receding resistance beyond the ligature, and the importance of leaving a good stump, we will avoid the dangers of secondary hemorrhage by observing greater care in the application of ligatures. Where it is desirable the application of a pair of heavy clamp forceps will prepare a bed for the ligature, or Doyen's instrument may be applied perfectly and the ligature may be discarded.

516 MARKET STREET.

